# AAPM Medical Physics Practice Guideline 3.a: Levels of supervision for medical physicists in clinical training

**DOI:** 10.1120/jacmp.v16i3.5291

**Published:** 2015-05-08

**Authors:** J. Anthony Seibert, Jessica B. Clements, Per H. Halvorsen, Michael G. Herman, Melissa C. Martin, Jatinder Palta, Douglas E. Pfeiffer, Robert J. Pizzutiello, Beth A. Schueler, S. Jeff Shepard, Lynne A. Fairobent

## Abstract

The American Association of Physicists in Medicine (AAPM) is a nonprofit professional society whose primary purposes are to advance the science, education and professional practice of medical physics. The AAPM has more than 8,000 members and is the principal organization of medical physicists in the United States.

The AAPM will periodically define new practice guidelines for medical physics practice to help advance the science of medical physics and to improve the quality of service to patients throughout the United States. Existing medical physics practice guidelines will be reviewed for the purpose of revision or renewal, as appropriate, on their fifth anniversary or sooner.

Each medical physics practice guideline represents a policy statement by the AAPM, has undergone a thorough consensus process in which it has been subjected to extensive review, and requires the approval of the Professional Council. The medical physics practice guidelines recognize that the safe and effective use of diagnostic and therapeutic radiology requires specific training, skills, and techniques, as described in each document. Reproduction or modification of the published practice guidelines and technical standards by those entities not providing these services is not authorized.

The following terms are used in the AAPM practice guidelines:

- Must and Must Not: Used to indicate that adherence to the recommendation is considered necessary to conform to this practice guideline.
- Should and Should Not: Used to indicate a prudent practice to which exceptions may occasionally be made in appropriate circumstances.

## 1. Introduction

The purpose of this practice guideline is to address the levels of supervision necessary for the clinical training of medical physics students, residents, and medical physicists‐in‐training. During the training of these individuals, it is often necessary or desirable for them to perform functions generally performed by Qualified Medical Physicists (QMPs). In such circumstances, the individuals performing these functions must be appropriately supervised and the scope of the functions to be performed must be carefully defined. This document does not address the use of medical physicist assistants or medical physics extenders (i.e., positions not intended to provide a path to becoming a QMP).

It is the responsibility of all individuals to be familiar with federal and state regulations regarding supervision that may take precedence over the recommendations.

## 2. Definitions

- **Competency** — The demonstrated ability to perform the medical physics‐related task or function independently.
- **Cosigning or Cosignature** — The process of obtaining a second signature or the formal process by which a report produced by a supervised individual is finalized by the supervising QMP. The QMP retains full responsibility for the trainee's work.
- **Formal Work Product** — A deliverable or outcome that must be produced as part of the clinical work to complete a project and achieve its objectives.
- **Medical Physicist‐in‐Training** — An individual who meets the requirements of certification, and is currently in the preparation to complete board certification in one or more of the subfields of medical physics.
- **Medical Physics Student** — An individual enrolled in a master's or doctoral degree‐granting program from an approved institution (e.g., program accredited by one of the organizations recognized by the Council on Higher Education Accreditation, or its successors), in medical physics, physics, or another relevant physical science or engineering discipline.
- **Medical Physics Resident** — An individual enrolled in a structured training program designed to educate and train to a level of competency sufficient to practice medical physics independently. This individual must have obtained a master's or doctoral degree in medical physics, physics, or another relevant physical science or engineering discipline. An individual enrolled in a Doctorate of Medical Physics (DMP) program also meets this definition of Medical Physics Resident while completing their clinical rotation requirements of the DMP program.
- **Qualified Medical Physicist** — As defined by AAPM Professional Policy 1.^(1)^
- **Supervision**: Oversight of and acceptance of responsibility by the QMP for the medical physics‐related work performed by a Trainee or Medical Physics Student:
  - **General Supervision** — The procedure is performed under a QMP's overall direction and control, but the QMP's presence is not required during the performance of the procedure. Under General Supervision, the training of the personnel who actually perform the procedure, and the maintenance of the necessary equipment and supplies, are the continuing responsibility of the QMP.
  - **Direct Supervision** — A QMP must exercise General Supervision and be present in the facility and immediately available to furnish assistance and direction throughout the performance of the procedure. It does not mean that the QMP must be present in the room when the procedure is being performed.
  - **Personal Supervision** — A QMP must exercise General Supervision and be present in the room during the performance of the procedure.^(2)^
- **Supervisor** — A QMP who oversees the medical physics‐related work of a supervised individual in a clinical environment.
- **Supervised Individual** — A medical physics student, resident or medical physicist‐in‐training performing medical physics‐related tasks under the direction of a QMP.
- **Trainee** — The term “trainee” is used to include medical physics residents and medical physicists‐in‐training.

## 3. Roles of the Medical Physics Student, Medical Physics Resident, or Medical Physicist‐in‐Training

- **Medical Physics Student**
  Medical physics students should be capable of performing basic medical physics tasks with appropriate training, such as the collection of X‐ray generator calibration for diagnostic / orthovoltage systems or linear accelerator depth dose data. The medical physics student is not expected to analyze or make decisions regarding the data, but may make comments or recommendations to the supervising QMP, and should be involved in the discussion of the data to further their education. If the medical physics student works on clinical medical physics tasks, it must be under the personal or direct supervision of a QMP, as deemed appropriate by the QMP.
- **Medical Physics Resident**
  Medical physics residents are expected to grow in the degree of responsibility and independence of clinical practice. Early in residency, the resident should have responsibilities similar to medical physics students. Personal supervision should be provided for the initial performance of tasks within the medical physics scope of practice.^(3)^ The resident, with experience and training, should progress to analysis of the data and performance of some functions under direct supervision. Late in the residency, the resident should be able to function largely as a QMP, with the supervisor balancing the transition from supervision toward independence of the resident during the residency program.
- **Medical Physicist‐in‐Training**

Medical physicists‐in‐training are expected to grow in the degree of responsibility and independence of clinical practice. With increasing experience, medical physicists‐in‐training should be able to function largely as a QMP, with the supervisor balancing the transition from supervision toward independence.

## 4. The Responsibilities of the Supervisor

Supervision is a responsibility that must not be undertaken lightly. The supervisor must assume professional responsibility for the medical physics‐related work done by the supervised individual. Should an untoward event occur, the professional reputation of the supervisor is at risk as if the supervisor had personally caused the incident.

Supervision of a trainee requires regular, high‐quality interactions between the supervisor and the trainee during which medical physics is practiced by the trainee under the guidance of the supervisor. As the trainee grows in professional maturity, it is appropriate for the supervisor to allow the trainee greater responsibility and autonomy, with the understanding that the supervisor must still review and cosign all formal work products of the trainee. Formal work products could include reports of machine calibrations, shielding designs, treatment plan reviews, patient‐specific quality assurance measurements, treatment record reviews, and equipment evaluations. By cosigning, the supervisor takes full professional responsibility for it as if it were the supervisor's own work. For “low‐level” work products as identified by the QMP supervisor, a supervised individual who has demonstrated competency through a formal work product may be able to independently sign off (the work product must describe the experience gained through personal, direct, and general supervision of the supervising QMP). Nevertheless, the supervising QMP retains full responsibility for the supervised individual's work for that specific work product, and all other work not similarly documented must be cosigned.

The supervisor must have a professional relationship with the supervised individual that allows the supervisor to observe the work of the supervised individual and to correct that work if necessary. The supervisor should not commit to the supervisory relationship if:

- The supervised individual and the supervisor work for different employers without a formal agreement outlining the supervisory relationship.
- The supervisor is unable to consistently provide the appropriate level of supervision.
- The supervisor has not committed to assuming full professional responsibility for the supervised individual's work.
- Except under documented extenuating circumstances (e.g., illness or job transition), or episodic training sessions, a supervisor must not supervise for the clinical training of medical physics:
  - more than two medical physics trainees at one time; and
  - more than two medical physics students at one time; and
  - more than three full‐time equivalents per supervisor. Supervised individuals who are part‐time in their clinical medical training are counted as their fractional FTE‐equivalent. For example, a medical physics student may only have 0.25 FTE in the clinic.

During a supervisor's absence, the supervisor is responsible for delegating the required supervision to the QMP providing coverage for the supervisor. There must be a clear description of what tasks may be performed independently by the supervised individual and under what circumstances a delegated QMP must cover the supervisory responsibility.

During a supervisor's absence from work, the supervised individual must only be allowed to perform tasks for which that individual has demonstrated and documented formal competency to work independently. Documentation of the supervised individual must be readily accessible in order to verify the ability to work independently. The exception is when the supervisor has a coverage arrangement with a colleague QMP during vacation time or other absence and that a coverage agreement specifically includes supervision of the supervised individual.

## 5. The Responsibilities of the Supervised Individual

The supervised individual must not perform clinical medical physics‐related tasks without appropriate supervision or documented competency by the QMP supervisor.

*For supervised individuals in states with professional licensure of medical physicists*: Currently, states that license medical physicists may require an individual working toward becoming a QMP to have a temporary or limited scope license to practice. If a supervised individual holds a temporary license in more than one subfield of medical physics, the supervisor must be fully licensed in all of the subfields the supervised individual is training toward (or in which the supervised individual holds a temporary license); otherwise, the supervised individual must secure more than one supervisor. However, the individual state mandates for supervision must be followed, as requirements may not be consistent in all states. It is the responsibility of the supervised individual to ensure that his or her supervisor(s) are licensed in the subfield at the time of the application for a temporary license.

## 6. A General Progression of Supervision for Trainees

There must be a progression from personal to general supervision for trainees. A supervision plan must be available and acknowledged by the trainee and the supervisor. Competency performance reviews should be completed and documented for each activity.

- **Supervision Plan**
  A supervision plan must be formally adopted and document well‐defined progression of levels of responsibility for trainees.
  As an example, a plan consisting of the following would meet the intent of this document:
  - Personal supervision for the first six months,
  - Direct supervision for the remainder of the first year, and
  - General supervision in the second year.
  Note that the supervisor is not relieved of the responsibility to review and cosign reports, and retains full responsibility for their content.
- **Considerations in Developing a Supervision Plan**
  Prior to determining the assignment of work, the facility must have a procedure consistent with the cosignature procedure for determining the conditions under which a supervised individual can practice.
  Care must be taken to provide sufficient supervision to all supervised individuals. It is essential that the QMP determine whether a supervised individual may visit a facility alone for a specific activity. The QMP must determine whether the QMP should also personally participate in the initial visit (or more as required) to a facility to address unanticipated issues. A supervised individual must not be sent alone to perform a procedure that is not in the scope of practice for the role of the supervised individual, or for which the supervised individual has not demonstrated formal competency.
  It is also essential that the QMP determine at what level a supervised individual may proceed from personal supervision to direct supervision and from direct supervision to general supervision. This may be task‐dependent, with higher‐level functions or critical patient‐care functions requiring more training and experience to be performed under personal or direct supervision than low‐level functions. Examples of such tasks are measurements performed on clinical teletherapy or imaging equipment to determine the output or beam characteristics. The progress plan with milestones to be achieved by the trainee should be documented.

## 7. Medical Physics Residency Programs

The Residency Program Director must be a QMP and have at least 5 years of full‐time experience beyond residency.^(4)^ All faculty members of a medical physics residency program should be fully qualified (including being licensed if practicing in a state with a medical physics licensure law) in the clinical activities that they supervise. The Residency Program Director may delegate day‐to‐day supervision of residents:

- to his or her colleagues on the faculty who are QMPs, or
- to other qualified faculty or staff members only if:
- ○ a QMP in the appropriate subfield of medical physics cosigns each item of work produced by the resident; and
- ○ the formal supervisor shares with the delegate the authority to observe and correct the work of the resident.

The Residency Program Director exercises General Supervision over all residents in the program. The Residency Program Director delegates responsibility for supervision to faculty members of the residency program by assignment of residents for a task or time period. Faculty members who have responsibility for supervision of a resident may further delegate authority for supervision to other faculty members who are QMPs for the tasks to be performed by the resident, or to faculty or staff members who are themselves medical physicists‐in‐training and are appropriately supervised by a QMP.

It is reasonable for one faculty member to be the formal supervisor of all of the residents, even more than two at a time, and to delegate day‐to‐day supervision to other qualified faculty members. Supervision cannot be delegated from a QMP to a resident; however, it is entirely appropriate for a more experienced resident to assist in the training of another resident. A QMP must supervise the training of one resident by another, and must cosign the joint work product of the two residents, who must also sign as residents. The supervising QMP is still ultimately responsible for the work of both residents.

## 8. Recommendations

Clinical practice environments must balance the need for competent staff with the need to train new professionals and provide services in a safe, yet cost‐effective, manner. The guidelines presented provide a framework for the appropriate supervision and scope of responsibilities for medical physics students and residents, and medical physicists‐in‐training.

Specifically:

- The supervisor must assume professional responsibility for the medical physics‐related work done by the supervised individual.
- A supervision plan must be formally adopted and document well‐defined progression of levels of responsibility for trainees.
- Except under documented extenuating circumstances (e.g., illness or job transition), a supervisor must not supervise:
- ○ more than two medical physics trainees at one time
- ○ more than two medical physics students

## ACKNOWLEDGMENTS

This guideline was developed by the Medical Physics Practice Guideline Task Group‐243 of the Professional Council of the AAPM.

TG‐243 Members:

J. Anthony Seibert, Chair, PhD, FAAPM, FACR, FSIIM

Jessica B. Clements, MS

Per H. Halvorsen, MS, FAAPM, FACR

Michael G. Herman, Ph.D., FAAPM, FACMP

Melissa C. Martin, MS, FAAPM, FACR, FACMP

Jatinder Palta, Ph.D., FAAPM, FACR, FASTRO, FACMP

Douglas E. Pfeiffer, MS, FAAPM, FACR

Robert J. Pizzutiello, Jr. MS, FAAPM, FACR, FACMP

Beth A. Schueler, Ph.D., FAAPM, FACR

S. Jeff Shepard, MS, FAAPM

Lynne A. Fairobent, AAPM Staff

AAPM Subcommittee on Practice Guidelines — AAPM Committee responsible for sponsoring the draft document through the process:

Russell Tarver, MS, Chair

Maria F. Chan, PhD, Vice‐Chair Therapy

Jessica B. Clements, MS

Dianna D. Cody, PhD, FAAPM,

Jonas D. Fontenot, PhD

Arthur J. Olch, PhD, FAAPM

Joann I. Prisciandaro, PhD, FAAPM

J. Anthony Seibert, PhD, FAAPM, FACR, FSIIM

S. Jeff Shepard, MS, FAAPM, Vice‐ Chair Imaging

Jennifer B. Smilowitz, PhD

Gerald A. White Jr., MS, FAAPM, FACR

Lynne A. Fairobent, AAPM Staff
